# Effect of long-chain inorganic polyphosphate treated with wheat phytase on interleukin 8 signaling in HT-29 cells

**DOI:** 10.5713/ab.21.0436

**Published:** 2022-01-05

**Authors:** Jeongmin An, Jaiesoon Cho

**Affiliations:** 1 Department of Animal Science and Technology, Konkuk University, Seoul 05029, Korea

**Keywords:** HT-29, Inflammatory Responses, Long-chain Inorganic Polyphosphate, Wheat Phytase

## Abstract

**Objective:**

This study was performed to investigate the potential effect of wheat phytase on long-chain inorganic polyphosphate (polyP)-mediated interleukin 8 (IL-8) signaling in an intestinal epithelial cell line, HT-29 cells.

**Methods:**

Cell viability and the release of the pro-inflammatory cytokine IL-8 in HT-29 cells exposed to polyP1150 (average of 1,150 phosphate residues) treated with or without wheat phytase were measured by the EZ-CYTOX kit and the IL-8 ELISA kit, respectively. Also, the activation of cellular inflammatory factors NF-κB and MAPK (p38 and ERK 1/2) in HT-29 cells was investigated using ELISA kits.

**Results:**

PolyP1150 negatively affected the viability of HT-29 cells in a dose-dependent manner. However, 100 mM polyP1150 dephosphorylated by wheat phytase increased cell viability by 1.4-fold over that of the intact substrate. Moreover, the 24 h exposure of cells to enzyme-treated 50 mM polyP1150 reduced the secretion of IL-8 and the activation of NF-κB by 9% and 19%, respectively, compared to the intact substrate. PolyP1150 (25 and 50 mM) dephosphorylated by the enzyme induced the activation of p38 MAPK via phosphorylation to 2.3 and 1.4-fold, respectively, compared to intact substrate, even though it had little effect on the expression of ERK 1/2 via phosphorylation.

**Conclusion:**

Wheat phytase could attenuate polyP1150-induced IL-8 release in HT-29 cells through NF-κB, independent of MAP kinases p38 and ERK. Thus, wheat phytase may alleviate inflammatory responses including hypercytokinemia caused by bacterial polyP infection in animals. Therefore, wheat phytase has the potential as an anti-inflammatory therapeutic supplement in animal husbandry.

## INTRODUCTION

Inorganic polyphosphate (polyP), a linear physiological polymer formed by structurally simple phosphoanhydride linkages, is largely categorized into short-chain polyP with fewer than 100 phosphate residues and long-chain polyP with up to about 1,000 phosphate residues [1,2]. Recently, the latter was reported to be responsible for the virulence of some bacteria. *Campylobacter jejuni*, a harmful food-poisoning bacteria in the poultry industry synthesized polyP through polyP kinase (PPK) and accumulated it within the polyP granules [1,3]. When bacterial infections occur in animals, polyP is released from the granules and acts as a chemotactic agent for immune cells [3,4]. In addition, polyP over-promotes macrophage differentiation to destroy the immunity of the host and induce strong inflammatory diseases [5,6]. In some studies, polyP upregulated the cytokine interleukin 11 (IL-11) in human osteosarcoma SaOS-2 cells and PPK stimulated the secretion of a well-known inflammatory response mediator interleukin 8 (IL-8) in human embryonic intestine INT407 cells [7,8]. Short-chain polyP induced a pro-inflammatory signaling response in human monocytic leukemia cells (THP-1) and primary human umbilical vein endothelial cells (HUVEC) by activating nuclear factor kappa-light-chain-enhancer of activated B cells (NF-κB) involved in various immune responses [9,10]. Many studies have reported that NF-κB was essential for the expression of IL-8 in various cells [11,12]. In addition, short-chain polyP could stimulate the activation of NF-κB and ERK and the production of IL-6 and TNF-α [13]. Many factors like bacterial polyPs and cellular regulators stimulate the activation of NF-κB, the main immune-related transcription factor [14,15]. ERK and p38, members of mitogen-activated protein kinase (MAPK), can affect NF-κB activation, which leads to interaction with diverse pro-inflammatory genes and facilitates gene expression [15]. In this study, long-chain polyP mainly released from bacteria stimulated NF-κB activity and induced IL-8 secretion, resulting in severe inflammatory symptoms such as diarrhea and fever in the host animals [16,17]. This eventually can bring huge commercial losses in animal husbandry [17].

The number of scientific reports on plant phytases showing the significant potential of feed supplements has increased considerably [18]. Wheat phytase, known as an unusual multiple inositol phosphate phosphatase possesses different phytate hydrolysis properties compared to fungal histidine acid phosphatase, the main constituent of commercial feed phytases [19]. Furthermore, wheat phytase non-specifically dephosphorylates long-chain polyP, which is essential to maintaining the pathogenicity of *C. jejuni*, *Escherichia coli*, and *Salmonella enterica serovar typhimurium*, the major causes of bacterial-borne diseases in animal husbandry [20].

Our hypothesis was that the dephosphorylation of long-chain polyP by wheat phytase may affect the expression of NF-κB and IL-8 associated with the inflammatory responses of the host cells, and that wheat phytase may increase cell viability by regulating hypercytokinemia in the cells. The mechanism of polyP-induced inflammation is still poorly understood. However, most of the studies have focused on short-chain polyP. The objective of the current study was to investigate the potential effect of wheat phytase on long-chain polyP-mediated IL-8 signaling in the intestinal epithelial cell line, HT-29.

## MATERIALS AND METHODS

### Reagents and cell culture

PolyP1150 with an average of 1,150 inorganic phosphate residues and wheat phytase were purchased from Kerafast (Boston, MA, USA) and Sigma-Aldrich (St. Louis, MO, USA), respectively. Both were reconstituted in endotoxin-free water (Sigma-Aldrich, USA). The human colorectal adenocarcinoma cell line, HT-29 was purchased from the American Type Culture Collection (ATCC, Manassas, VA, USA). The cells were grown in McCoy’s 5A medium (Gibco Life Technologies, Waltham, MA, USA) containing 10% fetal bovine serum and 1% penicillin-streptomycin solution (Gibco Life Technologies, USA). Cell cultures were maintained at 37°C in a humidified incubator with 95% air and 5% CO_2_.

### Cell viability assay

HT-29 cells were initially seeded onto a 96-well plate at a concentration of 2×10^4^ cells per well and cultured until 80% confluency. Different concentrations of polyP1150 (0, 10, 25, 50, 100, 200, and 400 mM) were added to the cells at 37°C for 48 h in a CO_2_ incubator. The cell viability was measured at optical density (OD) 450 nm using an EZ-CYTOX kit (DogenBio, Seoul, Korea) according to the manufacturer’s instructions.

### Effect of wheat phytase on cell viability

HT-29 cells were seeded onto a 96-well plate and cultured until 80% confluency. PolyP1150 (500 and 1,000 mM) was incubated at 37°C for 4 h with or without wheat phytase (42.9 mU/mL). After the incubation, the cells were treated with aliquots (10 μL) of the reaction mixtures at final concentrations of 50 and 100 mM polyP1150 at 37°C for 24 h in the CO_2_ incubator. Cell viability was analyzed at OD 450 nm using an EZ-CYTOX kit (DogenBio, Seoul, Korea).

### IL-8 assay

HT-29 cells were initially seeded onto 96-well plates and cultured until 80% confluency. PolyP1150 (10, 50, 250, and 500 mM) were treated at 37°C for 4 h with or without enzyme (42.9 mU/mL). After the incubation, aliquots (10 μL) of the reaction mixtures were added to the cells at final concentrations of 1, 5, 25, and 50 mM polyP1150 at 37°C for 6 h in a CO_2_ incubator. In addition, the cells were exposed to 50 mM polyP1150 with or without wheat phytase at 37°C for 1, 6, 12, and 24 h in a CO_2_ incubator. The levels of IL-8 secreted into the cell culture media were evaluated at OD 450 nm using a Cymax human IL-8 ELISA kit (Ab FRONTIER, Seoul, Korea) according to the manufacturer’s instructions.

### NF-κB assay

HT-29 cells were seeded onto a 96-well plate and cultured until 80% confluency. And we incubated polyP1150 at 37°C for 4 h with or without wheat enzyme (42.9 mU/mL). Aliquots (10 μL) of the reaction mixtures containing different concentrations of polyP1150 (10, 50, 250, and 500 mM) were added to the cells at final concentrations of 1, 5, 25, and 50 mM polyP1150 at 37°C for 6 h in a CO_2_ incubator. The cells were also exposed to 50 mM polyP1150 with or without wheat phytase at 37°C for 1, 6, 12, and 24 h in a CO_2_ incubator. Total human NF-κB p65 levels in the HT-29 cell lysates were measured at OD 450 nm using an NF-κB p65 (Total) InstantOne ELISA kit (Thermo Fisher Scientific, Waltham, MA, USA) according to the manufacturer’s instructions.

### p38 MAPK and ERK1/2 assay

HT-29 cells were seeded onto a 96-well plate and cultured until 80% confluency. Different concentrations of polyP1150 (10, 50, 250, and 500 mM), were incubated at 37°C for 4 h with or without phytase (42.9 mU/mL). Aliquots (10 μL) of the reaction mixtures were added to the HT-29 cells at final concentrations of 1, 5, 25, and 50 mM polyP1150 at 37°C for 6 h and incubated. The cells were also exposed to 50 mM polyP1150 with or without wheat phytase at 37°C for 1, 6, 12, and 24 h in a CO_2_ incubator. The activity of total and phospho p38 in the HT-29 cells was measured at OD 450 nm using the p38 (Total/Phospho) InstantOne ELISA kit (Thermo Fisher Scientific, USA), and the amount of total and phospho ERK 1/2 in the HT-29 cells was evaluated at OD 450 nm using the ERK 1/2 (Total/Phospho) InstantOne ELISA kit (Thermo Fisher Scientific, USA) according to the manufacturer’s instructions.

### Statistical analysis

Statistical significance between the groups was determined by a one-way analysis of variance with PROC general linear model (SAS 9.4, SAS Institute Inc, Cary, NC, USA) followed by Duncan’s multiple range test. The probability levels used for statistical significance were p<0.05. The results are expressed as the mean and standard error from three experiments.

## RESULTS

### Cell viability following polyP1150 treatment

**Figure 1:**
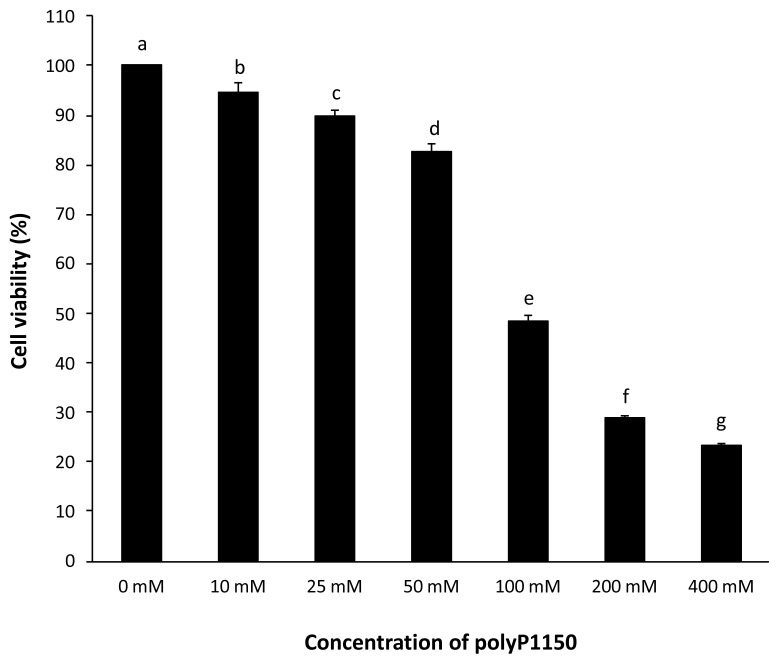
Cell viability of HT-29 cells exposed to long-chain inorganic polyphosphates. Data were expressed as mean and standard errors from three experiments. ^a–g^ Means lacking common superscripts differ significantly (p<0.05).

As shown in Figure 1, polyP1150 negatively affected the viability of HT-29 cells in a dose-dependent manner. In particular, there was a drastic change in cell viability between 50 mM and 100 mM substrate concentrations. At 50 mM, the cells retained 83% of the initial viability. However, the cell viability was severely decreased to 50% at 100 mM.

### Altered cell viability by polyP1150 treated with wheat phytase

**Figure 2:**
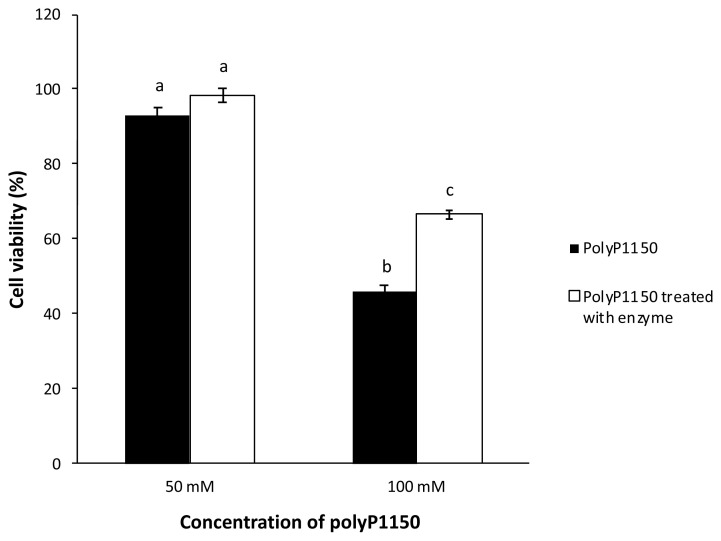
Cell viability of HT-29 cells exposed to long-chain inorganic polyphosphates treated with wheat phytase. Data were presented as mean and standard errors from three experiments. ^a–c^ Means lacking common superscripts differ significantly (p<0.05).

To some extent, polyP1150 dephosphorylated by wheat phytase alleviated the decrease in cell viability compared to intact polyP (Figure 2). In addition, 100 mM polyP1150 treated with the enzyme increased the cell viability by 1.4-fold over that of the intact substrate.

### Effect of long-chain inorganic polyphosphate added with wheat phytase on the release of IL-8 in HT-29 cells

**Figure 3:**
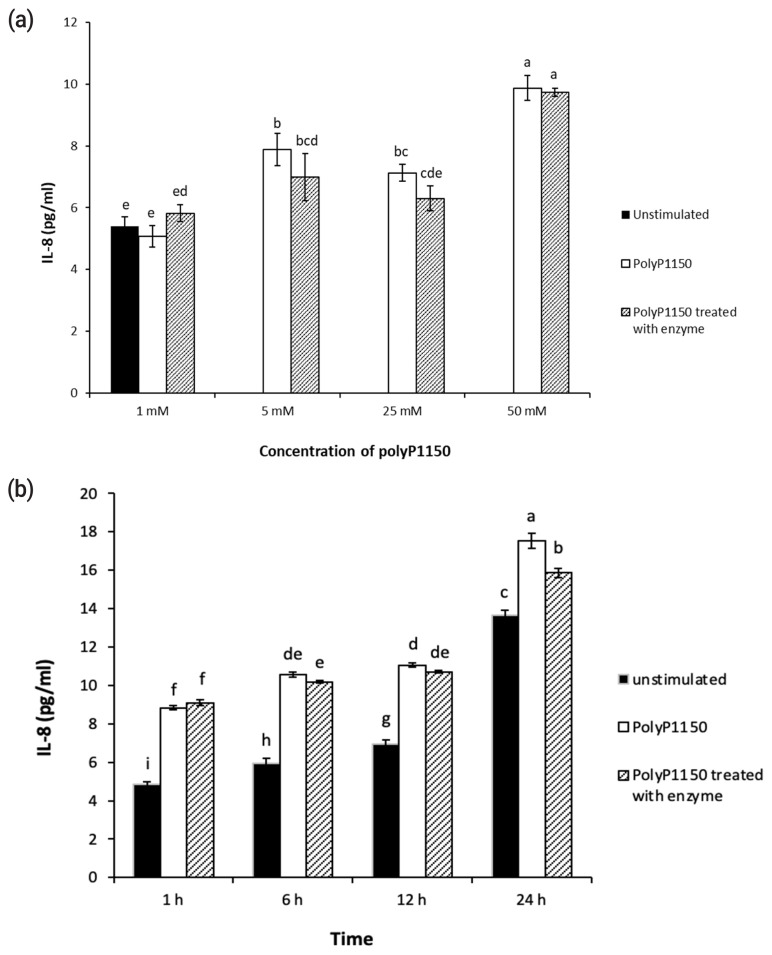
(a) Effect of various concentrations of long-chain inorganic polyphosphates treated with wheat phytase on interleukin 8 (IL-8) release in HT-29 cells. (b) Effect of long-chain inorganic polyphosphate (50 mM) treated with wheat phytase for different times on IL-8 release in HT-29 cells. Data were presented as mean and standard errors from three experiments. ^a–i^ Means lacking common superscripts differ significantly (p<0.05).

PolyP1150 failed to promote IL-8 secretion in HT-29 cells at a low concentration of 1 mM, and there was no effect of wheat phytase (Figure 3a). However, at 5, 25, and 50 mM, polyP1150 promoted IL-8 secretion from HT-29 cells to approximately 146%, 132%, and 183%, respectively, compared to unstimulated controls. In contrast, phytase-treated polyP1150 showed a tendency to decrease IL-8 release from HT-29 cells at 5 and 25 mM polyP1150 compared to intact polyp1150. Moreover, at 24 h exposure, 50 mM polyP1150 dephosphorylated by wheat phytase reduced the secretion of IL-8 from HT-29 cells by 9% compared to the intact substrate (Figure 3b).

### Effect of long-chain inorganic polyphosphate added with wheat phytase on the activation of NF-κB in HT-29 cells

**Figure 4:**
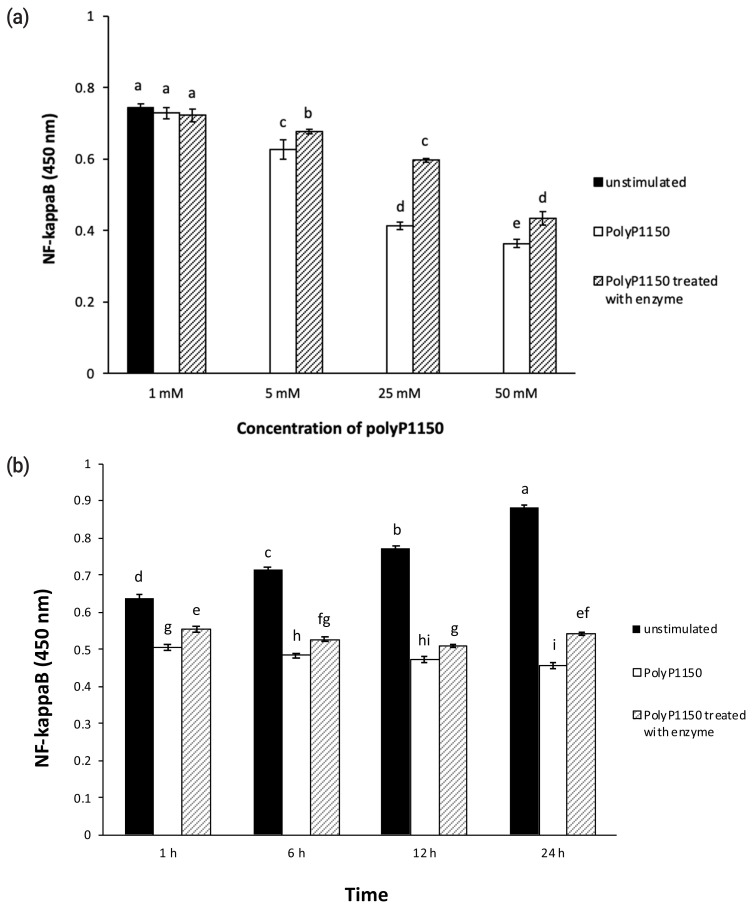
(a) Effect of various concentrations of long-chain inorganic polyphosphates treated with wheat phytase on nuclear factor kappa-light-chain-enhancer of activated B (NF-kB) activation in HT-29 cells. (b) Effect of long-chain inorganic polyphosphate (50 mM) treated with wheat phytase for different times on NF-kB activation in HT-29 cells. Data were presented as mean and standard errors from three experiments. ^a–i^ Means lacking common superscripts differ significantly (p<0.05).

A concentration of 50 mM polyP1150 enhanced the activation of NF-κB in HT-29 cells the most, by about 151% over that of unstimulated cells (Figure 4a). However, polyP1150 hydrolyzed by wheat phytase showed a tendency to reduce NF-κB activation in HT-29 cells compared to that of intact polyP1150. In addition, the activation of NF-κB was decreased by approximately 45% after exposure to wheat phytase-treated 25 mM polyP1150 compared to the intact substrate. As shown in Figure 4b, intact polyP1150 upregulated the activation of NF-κB in the cells in a time-dependent manner compared to unstimulated controls. However, 24 h exposure of cells to enzyme-treated polyP1150 reduced the activation of NF-κB by approximately 19% compared to cells treated with intact polyP1150.

### Effect of long-chain inorganic polyphosphate added with wheat phytase on the activation of p38 MAPK in HT-29 cells

**Figure 5:**
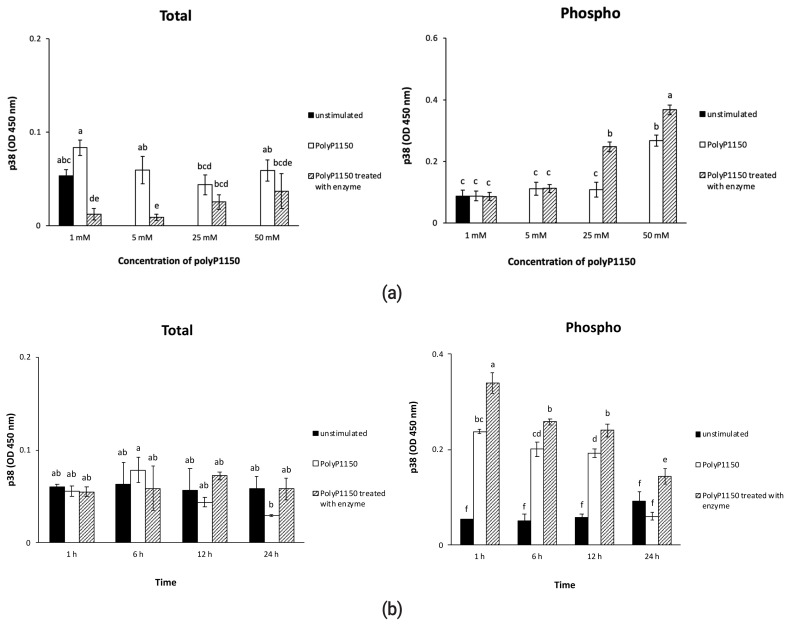
(a) Effect of various concentrations of long-chain inorganic polyphosphates treated with wheat phytase on total/phospho p38 activity in HT-29 cells. (b) Effect of long-chain inorganic polyphosphate (50 mM) treated with wheat phytase for different times on total/phospho p38 activity in HT-29 cells. Data were presented as mean and standard errors from three experiments. ^a–f^ Means lacking common superscripts differ significantly (p<0.05).

The total p38 MAPK levels were very low, whether the cells were exposed to polyP1150 or enzyme-treated substrate. However, 25 and 50 mM polyP1150 dephosphorylated by the enzyme induced the activation of p38 MAPK via phosphorylation 2.3 and 1.4-fold, respectively, compared to the intact substrates (Figure 5a). In addition, the phosphorylation of p38 MAPK was highest at 1 h exposure to 50 mM polyP1150 treated with the enzyme (Figure 5b).

### Effect of long-chain inorganic polyphosphate added with wheat phytase on the activation of ERK 1/2 in HT-29 cells

**Figure 6:**
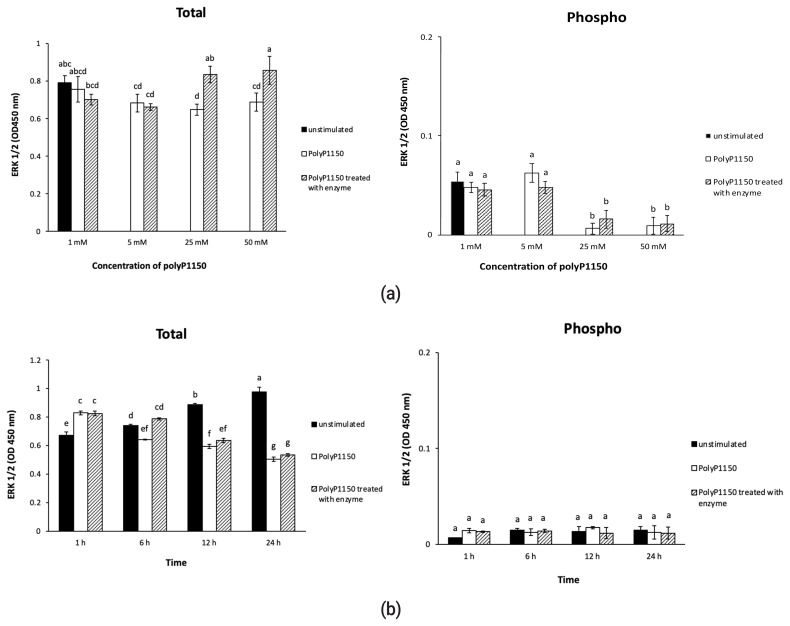
(a) Effect of various concentrations of long-chain inorganic polyphosphates treated with wheat phytase on total/phospho ERK 1/2 activityin HT-29 cells. (b) Effect of long-chain inorganic polyphosphate (50 mM) treated with wheat phytase for different times on total/phospho ERK1/2 activity in HT-29 cells. Data were presented as mean and standard errors from three experiments. ^a–g^ Means lacking common superscripts differ significantly (p<0.05).

As shown in Figures 6a and b, polyP1150 and wheat phytase-treated polyP1150 had little effect on the expression of ERK 1/2 via phosphorylation in HT-29 cells. However, 25 and 50 mM enzyme-treated polyP1150 increased the expression of total ERK 1/2 to 1.3 and 1.2–fold, respectively, compared to intact substrates. In addition, as HT-29 cells were exposed to 50 mM intact polyP1150 and enzyme-treated polyP1150 longer, the expression of total ERK 1/2 showed a tendency to decrease (Figure 6b).

## DISCUSSION

Inorganic polyphosphate existing within bacteria is released into animal cells and induces various mechanisms related to excessive inflammation [4]. Therefore, an inhibitor of polyP has the potential to be an anti-inflammatory agent [21]. However, because of the structural characteristics of polyP, anti-polyphosphate agents that inhibit polyP are not easily found [21].

In this study, pathogen-derived long-chain polyP1150 reduced the viability of HT-29 cells by more than 50% at 100 mM, indicating a fatal effect on the viability of HT-29 cells. PolyP, as an inflammatory material, reduced cell viability depending upon its concentration. And, polyP promoted the secretion of cytokines IL-11 and IL-8 [7]. But, the viability of HT-29 cells was increased after the application of wheat phytase to polyP1150, which was then used to treat the cells. Furthermore, polyP1150 that had been dephosphorylated by wheat phytase was less effective than intact polyP1150 at stimulating the secretion of the cytokine IL-8 in HT-29 cells. Indeed, polyP-mediated pro-inflammatory responses such as increased cytokine release were inhibited by the natural anticoagulant protease activated protein C (APC) in endothelial cells [9]. In this regard, wheat phytase alleviated the cytokine (IL-8) secretion induced by polyP in human epithelial cells and thus, has the potential to act as an anti-inflammatory enzyme like APC.

A number of previous studies showed that the activation of NF-κB was essential for IL-8 and the expression of many other pro-inflammatory genes [11,12]. NF-κB stimulates the expression of the IL-8 gene and immunoglobulin receptor in human amnion/cervical epithelial cells and HT-29 cells, respectively [12]. Also, sphingosine 1-phosphate induced IL-8 release mediated by NF-κB from airway epithelial cells [22]. In unstimulated cells, NF-κB is found in the cytoplasm as NF-κB is combined with inhibitory IkB proteins. Then, when the cells are stimulated and NF-κB is activated, NF-κB is released from the inhibitory protein and translocates to the nucleus where it activates transcription [23]. Some studies reported that polyP consisting of 45, 65, and 70 phosphate units activated NF-κB and elicited pro-inflammatory responses in human umbilical vein endothelial cells [9,10]. And the results obtained in our study revealed that like short-chain polyP (polyP45, 65, and 70), polyP1150 also stimulated NF-κB activation in the cells. After activation, NF-κB promoted inflammatory diseases in human intestinal epithelial cells [24]. According to a previous study, these inflammatory responses were alleviated by the adenosine A3 receptor through inhibition of the NF-κB signaling pathway in HT-29 cells [25]. Presumably, wheat phytase changed the mechanism of action of polyP on the HT-29 cells through the hydrolysis of polyP and attenuated the activation of NF-κB in HT-29 cells. And this change may have led to the decrease in cytokine (IL-8) release. The expression of IL-8 through the NF-κB pathway was mediated by p38 and ERK (MAPK) signaling in U937 cells [26]. Some studies have also indicated that MAPK transcription and NF-κB activation stimulated IL-8 release [15]. ERK, an atypical MAPK, interacts with c-JUN proteins and plays a critical role in AP-1 signaling that regulates the production of IL-8 [27]. However, our results showed that although p38 MAPK was phosphorylated in HT-29 cells, no clear interaction with the inflammatory responses caused by polyP1150 was seen. And there was no interaction between ERK and the activation of IL-8/NF-κB stimulated by polyP1150 in HT-29 cells. In a previous report, TNF-α-induced IL-8 secretion in HT-29 cells was mediated via the activation of ERK and p38, independent of NF-κB [28]. In contrast, in human airway epithelial cells, TNF-α-induced IL-8 secretion was regulated by NF-κB, independent of ERK [29].

The immune-regulating supplements currently used in animal husbandry to control bacterial polyP substances that cause destructive inflammation, do not act directly on the immune-stimulating mechanism of polyP. But, based on this study, the natural substance wheat phytase may regulate polyP1150-induced IL-8 release in HT-29 cells by NF-κB, independent of MAPKs such as p38 and ERK. And the implication of these findings is that wheat phytase can act directly on polyP and effectively inhibit pro-inflammatory responses. Therefore, this suggests that wheat phytase has the potential to regulate inflammatory disease caused by bacterial polyP, extending beyond nutritional effects in animal husbandry. Furthermore, wheat phytase may be a promising agent to safely modulate the excessive immune reaction of cells at the cellular level as an anti-inflammatory therapeutic supplement.
